# Intra- and inter-field strength reproducibility of deep-learning based real-time cardiac MRI cine sequences with breath hold and in free breathing

**DOI:** 10.1038/s41598-025-25154-6

**Published:** 2025-10-29

**Authors:** Lena-Maria Watzke, Ann-Christin Klemenz, Karolin K. Deyerberg, Benjamin Böttcher, Margarita Gorodezky, Mathias Manzke, Antonia Dalmer, Roberto Lorbeer, Danagul Zhexenova, Marc-André Weber, Felix G. Meinel

**Affiliations:** 1 Institute for Diagnostic and Interventional Radiology, Paediatric Radiology and Neuroradiology, University Medical Centre Rostock, Schillingallee 36, 18057 Rostock, Germany; 2 GE HealthCare, Munich, Germany; 3 Department of Radiology, Ludwig-Maximilian University, Munich, Germany; 4 University Medical Center, Astana, Kazakhstan

**Keywords:** Cardiac MR, Deep learning, Clinical imaging, Accelerated imaging, Free breathing, Reproducibility

## Abstract

To assess intra- and inter-field strength reproducibility of volumetric parameters using deep-learning-based real-time cardiac cine MRI during breath-hold (BH) and free-breathing (FB). In this prospective single-center study, 56 healthy adults underwent cardiac MRI at 1.5 T. Of these, 33 had a follow-up scan after 2–7 weeks, and 23 received an additional same-day scan at 3 T with the same protocol. Real-time cine sequences (1RR), including short-axis and 2-, 3-, and 4-chamber views, were acquired in BH and FB. Left ventricular volumes were analyzed using automated segmentation. Intra-class correlation coefficients (ICC) and subjective image quality (sIQ) were used to assess reproducibility. At 1.5 T, BH sequences showed significant differences in stroke volume (SV) and ejection fraction (EF), while FB sequences revealed only minor, clinically irrelevant SV variation. End-diastolic volume (EDV) and left ventricular (LV) mass showed excellent reproducibility (ICC > 0.93); end-systolic volume (ESV) and SV had good reproducibility (ICC 0.79–0.88). Inter-field comparisons revealed significant differences for EDV (BH), and for SV and EF (FB), though most parameters remained consistent. EDV, ESV, and LV mass showed excellent reproducibility (ICC > 0.90), and SV showed good to excellent agreement. Deep-learning-based real-time cine MRI provides good to excellent reproducibility of cardiac volumetric parameters across field strengths and breathing conditions.

## Introduction

Cardiac magnetic resonance imaging (CMR) is a fundamental diagnostic procedure for evaluating cardiac function and morphology^1,2^. The assessment of ventricular volumes as well as cardiac and valvular function is routinely performed using cine sequences^3^. For this purpose, images are acquired across multiple cardiac cycles and reconstructed into dynamic image series. Despite technological advances, long acquisition times, requiring breathing maneuvers, and motion artefacts remain significant challenges—particularly in cine sequences with multi-cycles of data acquisition, which are especially prone to artefacts in the presence of arrhythmias^4^.

The implementation of deep learning algorithms has led to substantial improvements in image acquisition and reconstruction processes^5,6^. Real-time cine imaging, where each slice is acquired within a single cardiac cycle, offers clear advantages in patients with arrhythmias by avoiding artefacts associated with multi-cycle acquisitions. However, its clinical use has historically been limited by a low signal-to-noise ratio, insufficient resolution and motion artefacts. Recent advances in deep learning-based reconstruction methods now enable k-space undersampling, resulting in enhanced image quality and accelerated acquisition times^7–10^. These developments also make free-breathing imaging possible, increasing patient comfort and making CMR more accessible to patients who are unable to hold their breath^11,12^ with reduced overall acquisition time, good to excellent image quality and acceptable volumetric results in healthy volunteers compared to sequences with breath hold^13^.

Building on these advancements, it is essential to evaluate the reproducibility of AI-accelerated imaging techniques, as it is a key factor in ensuring both diagnostic accuracy and scientific reliability. Despite the increasing clinical relevance of CMR, systematic studies on its reproducibility remain limited. This gap in research becomes particularly important when considering the various factors that can influence reproducibility, such as the magnetic field strength of the MRI system, inter-scan variability across different time points and patient-specific physiological differences^14^.

Therefore, the purpose of our study was to evaluate the intra- and inter-field strength reproducibility of volumetric parameter acquired with deep-learning based real-time cardiac cine sequences with breath holds (BH) and in free breathing (FB).

## Results

### Demographic characteristics

The final cohort of this prospective study included 50 healthy volunteers. Thrity-two participants underwent the repeated measurements at 1.5 T and 18 participants at 3 T. Detailed characteristics stratified by sex can be found in Table 1.

**Table 1:** Participants charateristics.

	Intra-field strength reproducibility at 1.5 T (n = 32)			Inter-field strength reproducibility at (1.5 T and 3 T, n = 18)		
	All participants (n = 32)	Men (n = 17)	Women (n = 15)	All participants (n = 18)	Men (n = 7)	Women (n = 11)
*Demographic parameters*						
Age [years]	41 (23–68)	42 (23–68)	40 (23–64)	46 (24–64)	46 (41–54)	45 (24–64)
Weight [kg]	77 (54–100)	84 (71–100)	70 (54–95)	84 (50–100)	93 (73–100)	72 (50–95)
Height [m]	1.75 (1.60–1.95)	1.83 (1.74–1.95)	1.70 (1.60–1.80)	1.73 (1.59–1.96)	1.80 (1.71–1.96)	1.69 (1.59–1.81)
BMI [kg/m^2^]	24.9 (18.0–32.9)	25.1 (20.2–28)	23.7 (18–32.9)	25.7 (19.4–33.2)	27.5 (22.5–31.8)	23.7 (19.4–33.2)
Heart rate [min^−1^]	67 (44–93)	64 (44–82)	68 (49–93)	73 (54–86)	75 (63–86)	68 (54–82)

### Intra-field strength reproducibility

#### Volumetric results

Volumetric analysis was performed using the short-axis stack acquired by the clinical reference sequence, the 1RR BH sequence and the 1RR FB sequence. The absolute volumetric results of the left ventricle for all sequences are shown in Table 2.

**Table 2:** Volumetric results for all left ventricular (LV) measurements from the first and second scans using the reference, 1RR breath-hold, and 1RR free-breathing sequences at 1.5 T.

	Intra-field strength reproducibility at 1.5 T (n = 32)											
	Reference (3RR)				1RR Breat hhold				1RR Free Breathing			
	1st measurement	2nd measurement	*p*-Value	ICC	1st measurement	2nd measurement	*p*-Value	ICC	1st measurement	2nd measurement	*p*-Value	ICC
LV EDV (ml)	161 (114; 245)	158 (114; 225)	0.291	0.962	165 (115; 256)	154 (119; 238)	0.166	0.936	157 (99; 233)	151 (113; 233)	0.911	0.932
LV ESV (ml)	64 (42; 105)	68 (40; 120)	0.155	0.815	65 (44; 108)	67 (41; 114)	0.304	0.882	66 (47; 109)	71 (43; 122)	0.082	0.792
LV SV (ml)	93 (72; 150)	88 (71; 148)	0.052	0.852	97 (71; 149)	91 (60; 142)	**0.021**	0.838	89 (48; 152)	89 (60; 132)	**0.014**	0.823
LV EF (%)	61 (46; 69)	57 (44; 66)	0.096	0.327	61 (46; 69)	57 (47; 68)	**0.024**	0.525	59 (42; 66)	55 (46; 67)	0.062	0.231
LV Mass (g)	100 (60; 162)	99 (63; 146)	0.125	0.962	100 (61; 159)	99 (63; 142)	0.197	0.975	100 (67; 162)	102 (61; 151)	0.400	0.986

Shown are the median, the range and the *p*-values by the Wilcoxon paired signed rank sum test and the Intraclass correlation coefficients (ICC) from two-way random- effects model. *LV *left ventricular, *EDV* End- diastolic volume, *ESV* End-systolic volume, *SV* stroke volume, *EF* ejection fraction.

P < 0.05 is considered as statistically significant and marked bold.

The largest differences between the first and second measurements were observed for SV and EDV. Notably, the 1RR BH sequence also demonstrated the most pronounced difference in EDV (10.1 ml) between both measurements.

While the 1RR BH generally exhibited slightly greater variability compared to the 3RR reference, with values fluctuating more between measurements, the 1RR FB sequence showed smaller variation especially in SV. For all other parameters, differences where comparable to the reference standard.

Statistical analysis of the 1.5 T cohort showed significant differences between the two measurements for SV in both the 1RR BH and 1RR FB sequences, and for EF in the 1RR BH sequence. All other parameters showed no statistically significant differences.

#### Correlation analysis

The ICCs indicate excellent reproducibility for EDV and LV mass across all sequences (ICC > 0.93). ESV and SV showed good reproducibility (ICC: 0.79–0.88). In contrast, EF demonstrated only poor to moderate reproducibility (ICC: 0.23–0.53) with the highest ICC for 1 RR BH (ICC=0.53). For a more detailed correlation analysis, the ICC values for all volumetric parameter can be found in Table 2.

#### Bland–Altman-analysis

**Fig. 1:**
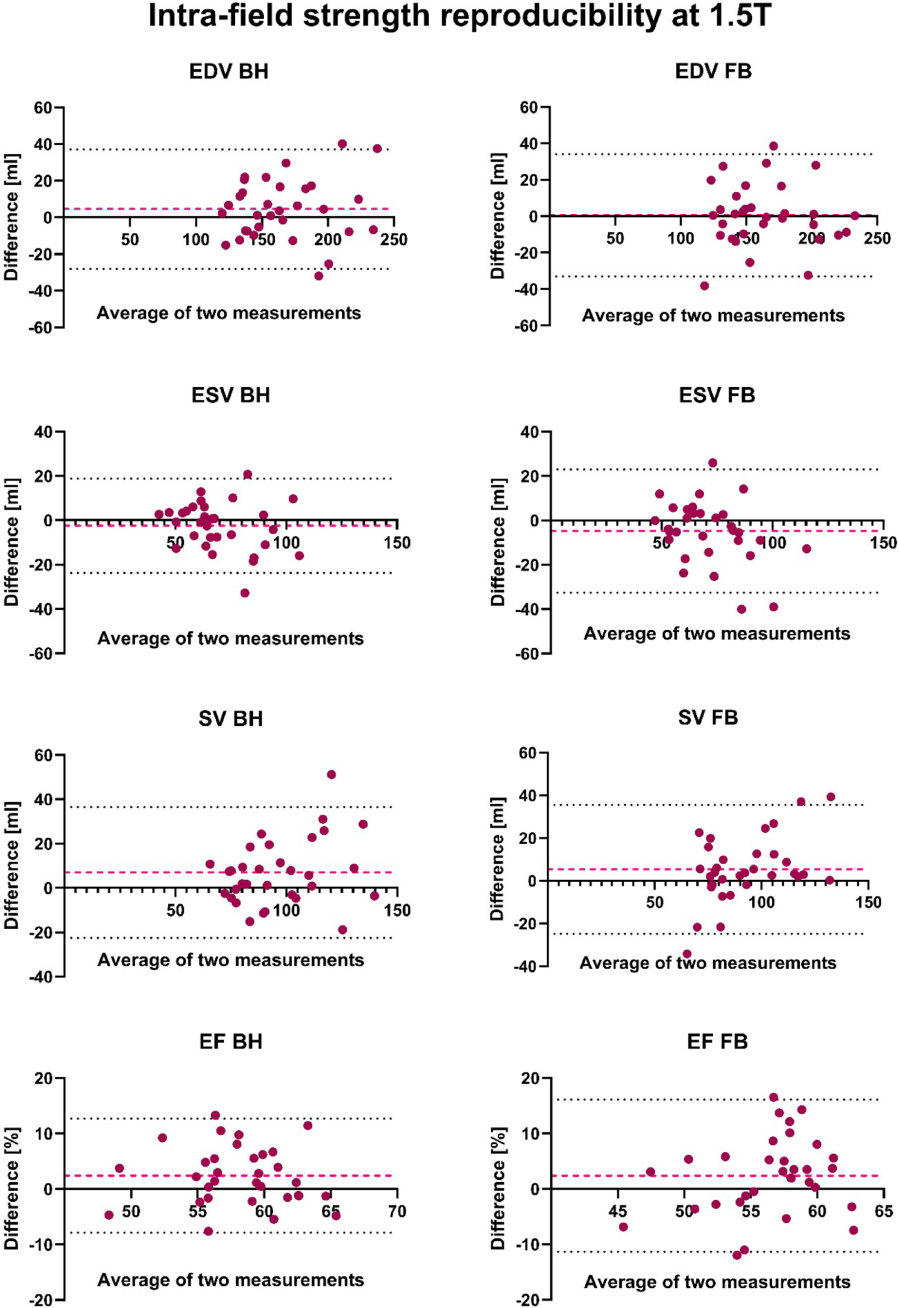
Bland–Altman Plots of all left ventricular (LV) volumetric parameters of the repetition measurement at 1.5 T. The second examination was performed on a different day with two to seven weeks in between. The red dashed line represents the bias (mean difference) between the two measurements. The black dotted lines indicate the limits of agreement (± 1.96 standard deviation of the difference).

The Bland-Altman analysis for intra-field strength reproducibility at 1.5 T revealed similar consistency between the BH and FB sequences. For EDV and SV, both sequences showed comparable wide limits of agreement (SV: –22.4 to 36.5 ml for BH vs. –24.8 to 35.6 ml for FB). ESV demonstrated slightly wider limits in the FB sequence (–32.5 to 23.0 ml) compared to BH (–23.7 to 18.8 ml). EF showed a consistent bias of 2.4% in both sequences, with slightly wider limits in FB. Full results are summarized in Table 3, with selected plots shown in Fig. 1.

**Table 3:** Bland–Altman Analysis of repeated measurements at 1.5 T. Shown are Bias, SD of bias and Limits of Agreement for all left ventricular (LV) parameters.

Bland–Altman analysis	Intra-field strength reproducibility at 1.5 T BH			Intra-field strength reproducibility at 1.5 T FB		
Parameter	Bias	SD of bias	95% Limits of agreement	Bias	SD of bias	95% Limits of agreement
LV EDV (ml)	4.5	16.6	− 28.1–37.1	0.6	17.1	− 33.0–34.2
LV ESV (ml)	− 2.5	10.6	− 23.7–18.8	− 4.8	14.2	− 32.5–23.0
LV SV (ml)	7.0	15.0	− 22.4–36.5	5.4	15.4	− 24.8–35.6
LV EF (%)	2.4	5.3	− 7.9–12.7	2.4	7.0	− 11.3–16.1

#### Differences between Measurements

**Fig. 2:**
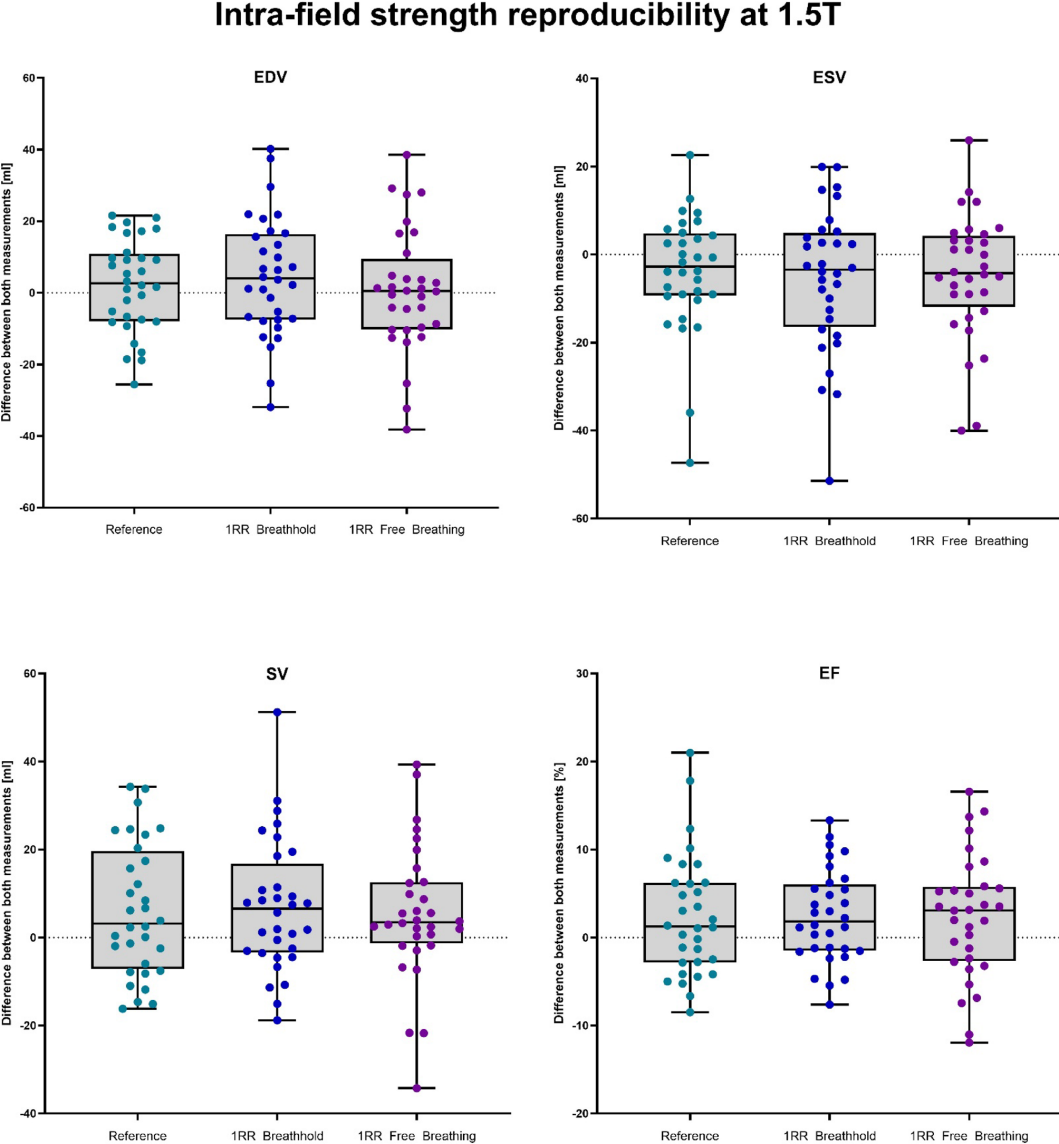
Boxplots of the difference of the volumetric parameters between the first and the second MRI examination of 32 healthy volunteers at 1.5 T. The second examination was performed on a different day with two to seven weeks in between.

For a comparison of the fluctuations of quantitative CMR values, the difference between repeated measurements at 1.5 T of all sequences was compared and is displayed in Fig. 2. For EDV the median differences between the repetitions were 2.6 ml, 4.0 ml and 0.5 ml for the reference, 1RR BH and 1RR FB, respectively. The ESV showed the maximum difference at the 1RR FB sequence with a median value of − 4.3 ml, whereas reference (median EDV = − 2.8 ml) and the 1RR BH (median EDV = − 0.7) resulted in lower differences between the two measurements. For SV and EF the differences of the reference where smallest (3.2 ml and 1.3%), but 1RR FB showed a comparable median for SV with 3.5 ml and 1RR BH for EF with 1.8%. The variability was in general slightly higher in 1RR BH and 1RR FB results compared to the reference.

### Inter-field strength Reproducibility

#### Volumetric analysis

The largest difference between the two measurements was observed for EDV in the 1RR BH sequence (10.3 ml), where also the greatest variation in the range was observed (95.1 ml to 190 ml for EDV). However, the 1RR BH sequence was overall the sequence with the greatest variability and the largest range differences. In contrast, the 1RR FB sequence demonstrated the smallest differences in EDV, SV, and LV Mass. The EF value showed relatively constant values across all sequences; however, the range of the 1RR BH sequence (from 45 to 70%) was wider than that of the 1RR FB sequence (from 44 to 65%). Notably, the 3RR reference was the most stable for EF, with a minimal difference of only 0.5% and the narrowest range (from 50 to 65%).

Statistical analysis revealed a significant difference between the two measurements for EDV in the 1RR BH sequence (*p* = 0.011), and for SV in the 1RR FB sequence (*p* = 0.010). Additionally, EF showed a significant difference in the 1RR FB sequence (*p* = 0.031). All other parameters in all sequences did not reach statistical significance.

#### Correlation analysis

In the inter-field strength comparison, EDV, ESV and LV mass demonstrated excellent reproducibility across all sequences (ICC >0.90). SV showed good reproducibility for the BH (ICC = 0.73) and FB (ICC = 0.82) sequences and excellent reproducibility for the Reference sequence (ICC = 0.91). EF demonstrated only poor to moderate reproducibility (ICC = 0.53–0.65), with lower ICC observed in the BH sequence (ICC: 0.53) compared to the FB sequence (ICC: 0.65). All ICC values are also summarized in Table 4.

**Table 4:** Volumetric results for all left ventricular (LV) measurements from the first (1.5 T) and second (3 T) scans using the reference, 1RR breath-hold, and 1RR free-breathing sequences.

	Inter-field strength reproducibility (1.5 T and 3 T, n = 18)											
	Reference (3RR)				1RR Breathhold				1RR Free Breathing			
	1st measurement	2nd measurement	*p*-Value	ICC	1st measurement	2nd measurement	*p*-Value	ICC	1st measurement	2nd measurement	p-Value	ICC
LV EDV (ml)	148 (101; 190)	139 (95; 185)	0.078	0.975	146 (96; 189)	136 (92; 180)	**0.011**	0.918	146 (96; 180)	140 (97; 183)	0.286	0.951
LV ESV (ml)	62 (36; 96)	57 (33; 87)	0.586	0.927	61 (36; 105)	54 (33; 85)	0.112	0.900	57 (41; 100)	62 (40; 105)	0.112	0.913
LV SV (ml)	88 (58; 118)	83 (62; 113)	0.231	0.905	86 (60; 106)	81 (48; 100)	0.122	0.727	81 (51; 106)	79 (48; 95)	**0.010**	0.815
LV EF (%)	59 (50; 65)	59 (51; 65)	0.948	0.631	58 (45; 65)	59 (46; 65)	0.420	0.527	56 (44; 70)	54 (43; 62)	**0.031**	0.653
LV Mass (g)	87 (60; 141)	85 (63; 139)	0.500	0.994	83 (60; 137)	86 (56; 135)	0.586	0.987	83 (59; 130)	83 (58; 136)	0.557	0.983

Shown are the median, the range and the p-values by the Wilcoxon paired signed rank sum test and the Intraclass correlation coefficients (ICC) from two-way random- effects model. *LV* left ventricular, *EDV* End- diastolic volume, *ESV* End-systolic volume, *SV* stroke volume, *EF* ejection fraction.

P < 0.05 is considered as statistically significant and marked bold.

#### Bland–Altman-Analysis

**Fig. 3:**
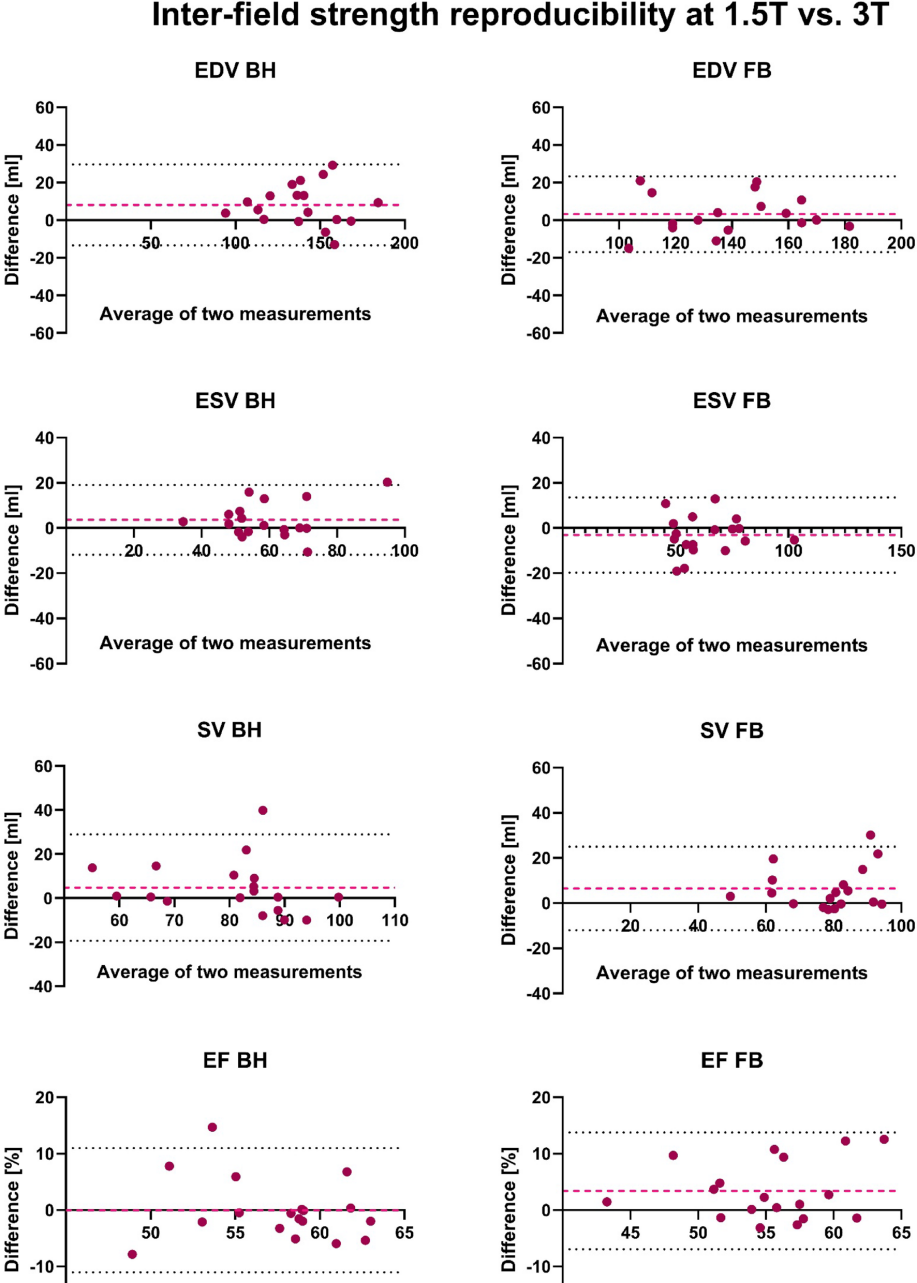
Bland–Altman Plots of all left ventricular (LV) volumetric parameters of the repetition measurement at 3 T. The second examination was performed on the same day. The red dashed line represents the bias (mean difference) between the two measurements. The black dotted lines indicate the limits of agreement (± 1.96 standard deviation of the difference).

The Bland-Altman analysis revealed smaller bias and narrower limits of inter-field strength agreement for the FB sequence compared to the BH sequence, particularly for LV EDV and SV. The limits of agreement for both LV EDV and LV SV were narrower for FB (–16.9 to 23.4 ml and –12.0 to 24.9 ml, respectively) compared to BH (–13.4 to 29.6 ml and –19.4 to 28.9 ml, respectively). In contrast, LV ESV showed slightly wider limits of agreement in the FB sequence. Detailed results are provided in Table 5, with selected parameter visualizations presented in Fig. 3.

**Table 5:** Bland–Altman Analysis of repeated measurements at 3 T.

Bland–Altman analysis	Inter-field strength reproducibility (1.5 T and 3 T) BH			Inter-field strength reproducibility (1.5 T and 3 T) FB		
Parameter	Bias	SD of bias	95% Limits of agreement	Bias	SD of bias	95% Limits of agreement
LV EDV (ml)	8.1	11.0	− 13.4–29.6	3.2	10.28	− 16.9–23.4
LV ESV (ml)	3.6	7.9	− 11.9–19.1	− 3.2	8.5	− 19.8–13.5
LV SV (ml)	4.7	12.3	− 19.4–28.9	6.5	9.4	− 12.0–24.9
LV EF (%)	-0.03	5.6	− 11.0–11.0	3.4	5.3	− 7.0–13.7

Shown are Bias, SD of bias and Limits of Agreement for all left ventricular (LV) parameters.

#### Differences between measurements

**Fig. 4:**
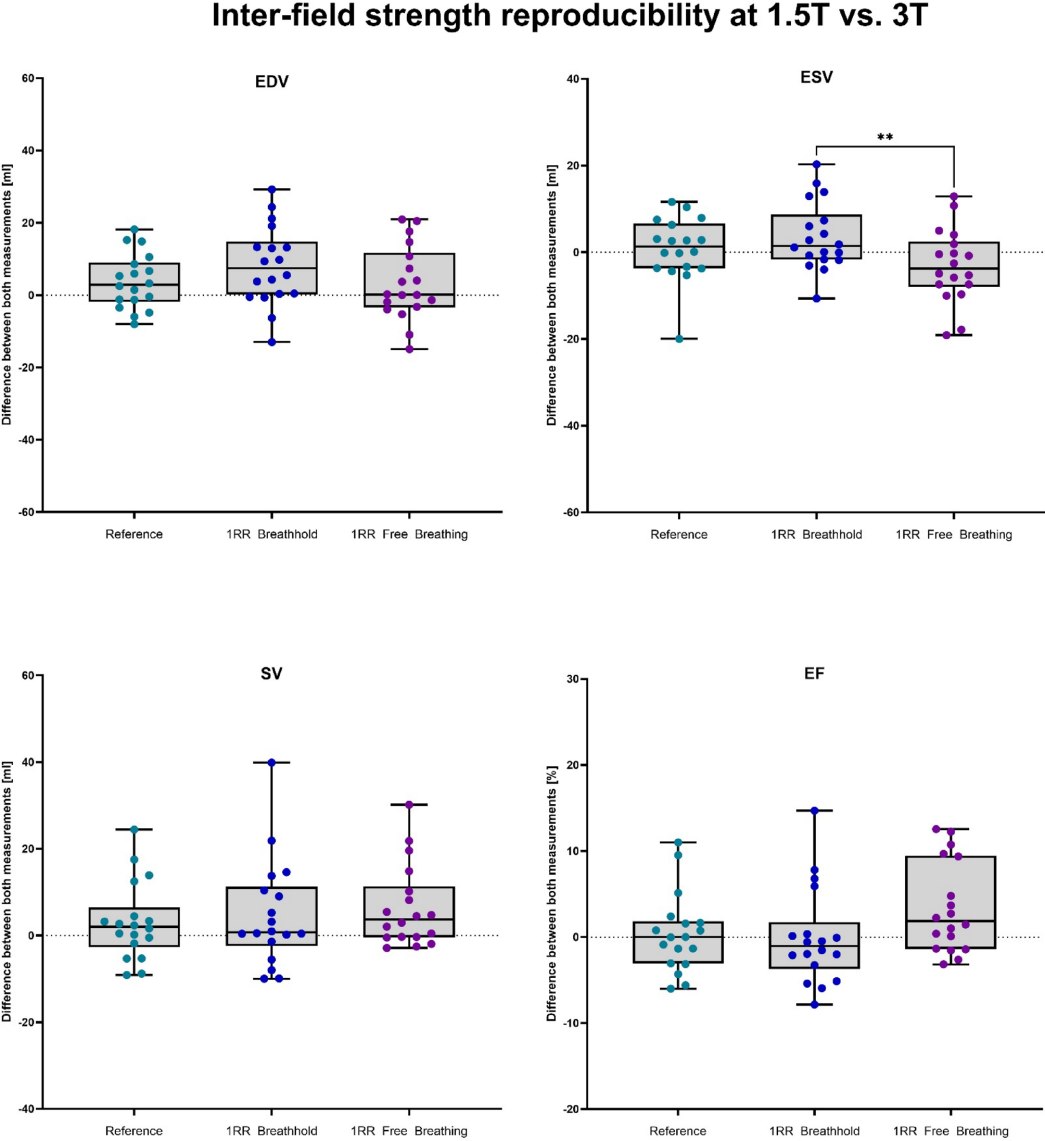
Boxplots of the difference of the volumetric parameters of 18 healthy volunteers between the first examination performed at 1.5 T and the second MRI examination at 3 T. The second examination was performed on the same day.

The measurements of the same volunteers on a 1.5 T and a 3 T MRI system on the same day overall showed smaller differences compared to repeated measurement at 1.5 T on different measurement days. Volumetric values of the reference sequences showed median differences of 2.9 ml, 1.4 ml, 2.0 ml and 0% for EDV, ESV, SV and EF, respectively. The 1RR BH showed highest deviations for EDV with a median of 7.5 ml, followed by ESV (median = 1.5 ml), EF (median = − 1.1%) and SV (median = 3.7 ml). The differences of the 1RR FB volumetric parameter resulted in a significant difference to the ESV differences of the 1RR BH sequence with a median of − 3.7 ml. The differences at EDV were only minor with 0.2 ml between repeated measurements whereas differences for SV (median = 3.7 ml) and EF (median = 1.9%) reached largest deviations at the 1RR FB sequence compared to the reference and the 1RR BH. A visualisation of the data can be found in Fig. 4.

### Subjective image quality for all measurements

**Fig. 5:**
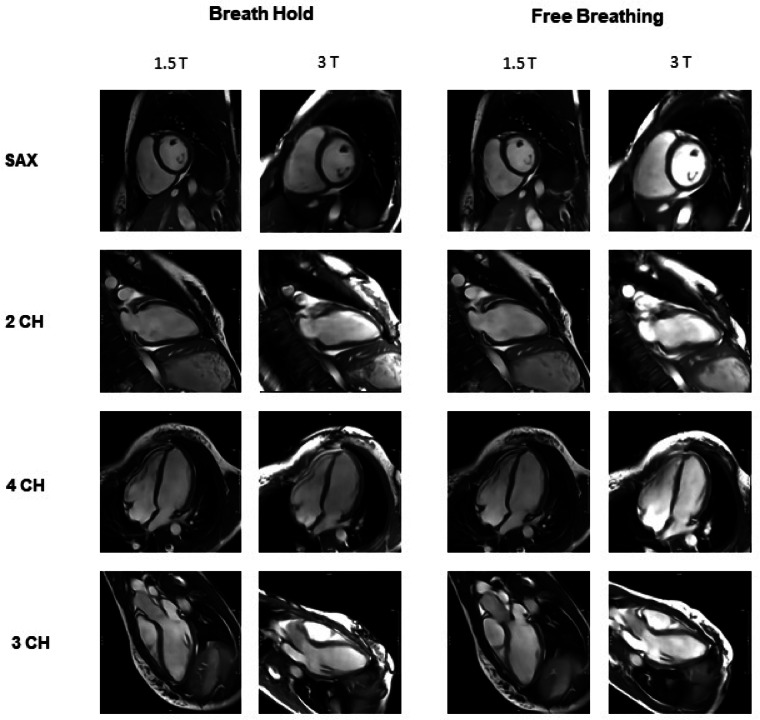
Image examples of the 1RR breath hold and 1RR free breathing sequences at both 1.5 T and 3 T for the long-axis views (2-Chamber (2 CH), 3-Chamber (3 CH) and 4-Chamber (4 CH)) and a midventricular slice from the short-axis stack (SAX).

Subjective image quality was generally rated higher at 1.5 T compared to 3 T, with the largest differences observed in breath-hold cine sequences. The mean overall score for the 2-, 3-, and 4-chamber views as well as the short-axis stack at 1.5 T with breath-hold were 4.5 (range: 4–5), 4.7 (range: 4–5), 4.7 (range: 4–5), and 4.3 (range: 4–5), respectively. In contrast, the corresponding 3 T sequences yielded lower ratings of 3.3 (range: 2.5–4), 3.5 (range: 2.5–4), 3.3 (range: 2.5–4), and 3.3 (range: 3–3.5), respectively. For free-breathing sequences, 1.5 T also achieved higher scores: average values were 4.2 (range: 3.5–4.5), 4.3 (range: 3.5–4.5), 4.5 (range: 4–5), and 4.1 (range: 3.5–5) for the 2-, 3-, 4-chamber views and the short-axis stack. At 3 T, the same free-breathing sequence achieved lower mean scores of 3.7 (range: 3.5–4), 3.8 (range: 3.5–4), 3.8 (range: 3.5–4), and 3.7 (range: 3.5–4.3), respectively. The main differences between 1.5 and 3 T, as well as the lowest scores overall, were consistently seen in the contrast assessment across all cardiac planes. Detailed ratings of the subjective image quality can be found in Table 6. Image examples of the 1RR BH and 1RR FB sequences at both 1.5 T and 3 T for the long-axis views and the midventricular short-axis view are shown in Fig. 5.

**Table 6:** Median values of subjective image quality of all heart planes in the categories contrast, blurring and artefacts.

	Subjective image quality of breath hold (BH) sequences			Subjective image quality of free breathing (FB) sequences		
	1.5 T	3 T	*p*-Value	1.5 T	3 T	*p*-Value
2CH 1RR	**4.5**	**3.3**	** < 0.001**	**4.2**	**3.7**	**0.002**
Contrast	5.0	2.5	** < 0.001**	4.5	4.0	0.083
Sharpness	4.0	4.0	0.114	3.5	3.5	**0.046**
Absence of Artefacts	4.5	4.0	**0.001**	4.5	4.0	0.068
3CH 1RR	**4.7**	**3.5**	** < 0.001**	**4.3**	**3.8**	**0.005**
Contrast	5.0	2.5	** < 0.001**	4.5	4.0	**0.001**
Sharpness	4.0	3.5	** < 0.001**	3.5	3.5	**0.024**
Absence of Artefacts	5.0	4.0	** < 0.001**	4.5	4.0	0.163
4CH 1RR	**4.7**	**3.3**	** < 0.001**	**4.5**	**3.8**	** < 0.001**
Contrast	5.0	2.5	** < 0.001**	5.0	4.0	** < 0.001**
Sharpness	4.0	3.5	**0.003**	4.0	3.5	**0.006**
Absence of Artefacts	5.0	4.0	**0.006**	5.0	4.0	**0.001**
Sax 1RR	**4.3**	**3.3**	** < 0.001**	**4.1**	**3.7**	** < 0.001**
Contrast	5.0	3.0	** < 0.001**	5.0	4.3	** < 0.001**
Sharpness	4.0	3.0	** < 0.001**	3.5	3.5	**0.014**
Absence of Artefacts	4.5	3.5	** < 0.001**	4.0	3.5	**0.009**

Displayed are the individual values of 2- chamber (2CH), 3- chamber (3CH), 4- chamber (4CH) and short axis stack (Sax) for repeated measurement at 1.5 T and 3 T of breath hold (BH) and free breathing (FB) sequences. Corresponding p-values are provided.

P < 0.05 is considered as statistically significant and marked bold.

## Discussion

Previous studies have primarily investigated the accuracy and image quality of deep learning-based CMRI sequences in comparison with conventional sequences^11^, but without analyzing repeatability. However, the evidence on reproducibility across repeated measurements for deep learning-based cine MRI under free-breathing conditions is very limited. This study addresses this issue by providing systematic data on test–retest stability under real-life conditions for the first time, through repeated measurements of DL-based real-time cine imaging. This is essential for the clinical validation of AI-based imaging. Our findings indicate that the repeatability of measurements under free-breathing conditions is comparable to that of the conventional breath-hold technique.

DL-based accelerated, undersampled cine sequences from the same vendor were already used in previous studies^8,10,11,15^. They revealed slightly reduced but still good to excellent image quality and accurate volumetric results.

While all of these studies^7,16–18^ demonstrate good agreement between deep learning (DL)-based cine MRI and conventional sequences, none of them systematically analyzed test–retest reproducibility under conditions that are representative of a typical clinical setting. While a few studies have examined test–retest reproducibility in cardiac MRI, these studies typically use conventional sequences or focus on image quality rather than volumetric parameters^19^. Studies such as those by^20^ have also examined the reproducibility of cardiac cine MRI, but these were based on a very small cohort (n = 5) and did not use deep learning or free breathing. Another study^21^ determined test–retest reproducibility of cardiac MRI with a conventional sequence in 20 healthy volunteers (in addition to the reproducibility of echocardiography). The results of the absolute difference between both measurements were comparable to ours. While good agreement was also found for volume parameters in these studies, statistical significance was limited due to the small sample size. Our study provides systematic data on the reproducibility of volumetric parameters under realistic conditions for the first time, including free breathing and scanner changes, in a larger healthy cohort. Despite significantly shorter acquisition times and the use of highly accelerated, deep learning (DL)-based real-time cine sequences, we were able to demonstrate consistently high to excellent intraclass correlation coefficients (ICC > 0.90) for end-diastolic volume (EDV), end-systolic volume (ESV) and stroke volume (SV)—both under breath-hold and free-breathing conditions. Notably, the high reproducibility under free breathing is especially important, as it significantly enhances the clinical applicability of this method for patients with impaired breathing ability.

We found good to excellent reproducibility for all volumetric parameters except for ejection fraction. Since the EF is a calculated parameter (EF = (EDV-ESV)/EDV) the differences for the ICC calculation is triplicated and therefore the ICC shows only moderate values for the EF. Furthermore we investigated a cohort of healthy volunteers. While there are relevant differences in cardiac volumes and myocardial mass between healthy individuals, all have a normal ejection fraction without meaningful differences. We would except to see a higher intra-class correlation coefficient for ejection fraction in a patient cohort with clinically meaningful differences in ejection fraction.

In our study, the overall reproducibility of volumetric results with deep-learning based real-time cine sequences was in a similar range as seen for an accelerated cine sequence acquired over 3 cardiac cycles (3RR), that has been previously validated against conventional multi-cycle cine imaging^11^. This suggests that the residual test–retest variability observed in our study may largely reflect true physiological fluctuations rather than measurement error due to the highly accelerated sequence technique. Fitting with this hypothesis is our observation that variability was greater for different examinations performed on the same 1.5 T system on different days than for examinations performed with different field strengths on the same day. Some outliers with higher differences between both measurements were detected. This was due to inconsistencies in the number of segmented slices during the fully automated segmentation. To eliminate the subjective influence during the volumetric post-processing, the fully automated segmentation was chosen and therefore those discrepancies of different segmented slices was not corrected, leading to increased volumetric differences in some cases.

In our analysis of subjective image quality, we found that image quality of deep-learning based real-time cine sequences were significantly superior for 1.5 T compared to 3 T. The difference was largely driven by higher ratings for “image contrast” and “absence of artefacts” at 1.5 T. While it is well known that SSFP based cardiac cine sequences are highly susceptible to artefacts at 3 T^22^, we were surprised to see inferior perceived image contrast at 3 T. Previous publications have shown that 3 T can improve signal-to-noise and contrast-to-noise ratio compared to 1.5 T^22–24^. In our study, both scanners provide a good signal-to-noise and contrast-to-noise ratio. The worse image quality on the 3 T could be explained by stronger susceptibility effects causing more artefacts and protocol being matched to the 1.5 T protocol. To improve image quality on 3 T an optimised protocol for that field strength could be used. Overall, our results confirm that DL-based cine-MRI under free-breathing conditions can be considered a valid clinical alternative to conventional breath-hold sequences in terms of both image quality and volumetric accuracy.

This study is an important step towards developing a robust, accelerated and patient-friendly cardiac MRI protocol. Its clinical relevance lies in the high reproducibility and comparability of volume parameters such as EDV, SV and EF at different time points, on different scanners and at different field strengths—a key aspect for follow-up examinations in chronic heart disease.

DL-based cine MRI could even be more robust than classic breath-hold techniques especially in cases of patient incompliance and arrhythmia. The ability to reliably acquire these parameters even under free breathing is crucial, as it makes the technology applicable to a wider range of patients, even children^25^.

The proven reproducibility at 1.5 T and 3 T also forms the basis for standardising CMR protocols and promoting comparability in multicentre studies. In the long term, further development of AI-supported imaging could accelerate diagnostics further, optimise the clinical workflow and thus improve patient care. Further studies with larger and more diverse cohorts are required for validation, particularly in paediatrics and in different patient groups.

This study has limitations. First, we evaluated accelerated sequences from one vendor at two magnet field strengths in a single center study. Secondly, all data were obtained from healthy adult volunteers, and there is a lack of fully sampled data from patients with heart disease or pediatric subjects. We would expect that reproducibility in free breathing may be superior to breath hold sequences in those patients, who are unable to hold their breath. Furthermore, no manual improvement of the segmentation—as commonly used in daily clinical routine—was performed to verify the accuracy of the results. Including such a reference could have provided an additional layer of error control and enhanced the reliability of the measurements. We only investigated intra-field strength reproducibility at 1.5 T and inter-field strength reproducibility between 1.5 and 3 T scanners but not intra-field strength reproducibility at 3 T. Furthermore the individual cohorts for inter- and intra-field strength reproducibility could have been larger.

## Conclusion

Deep-learning based real-time cine CMR sequences both with breath hold and in free breathing show good to excellent reproducibility regarding the volumetric parameters. Differences between different examinations performed on the same 1.5 T system examination on different days were greater than the influence of differences between examinations performed with different field strengths on the same day.

## Material and methods

### Ethical approval and participants selection

The present prospective single-centre cohort study was approved by the responsible institutional review board (University medical center Rostock) and all research was performed in accordance with relevant guidelines/regulations and in accordance with the Declaration of Helsinki. Prior to participation, all individuals provided written informed consent. Exclusion criteria included pregnancy, general contraindications to MRI (e.g. non-MRI-compatible implants or severe claustrophobia) and pre-existing medical conditions with potential impact on cardiac morphology or function, including cardiovascular diseases, chronic pulmonary disorders, arterial hypertension, and diabetes mellitus.

A cohort of 56 heart-healthy adult participants (aged over 18 years) were recruited prospectively from the staff at the University Medical Center. Six participants did not complete all MRI sequences or did not show up for the second MRI appointment. The data of these participants were excluded from the subsequent analysis.

### Study design and MRI protocol

All participants initially underwent a first cardiac MRI examination performed on a 1.5 T MRI system (Signa Artist, GE HealthCare, Solingen, Germany). Following the initial scan, all participants underwent a second MRI examination.

For evaluation of the reproducibility of the sequences and to account for physiological variability over time 33 participants repeated the scan on the same 1.5 T system on a separate day, with an interval of two and seven weeks between the two sessions.

**Fig. 6:**
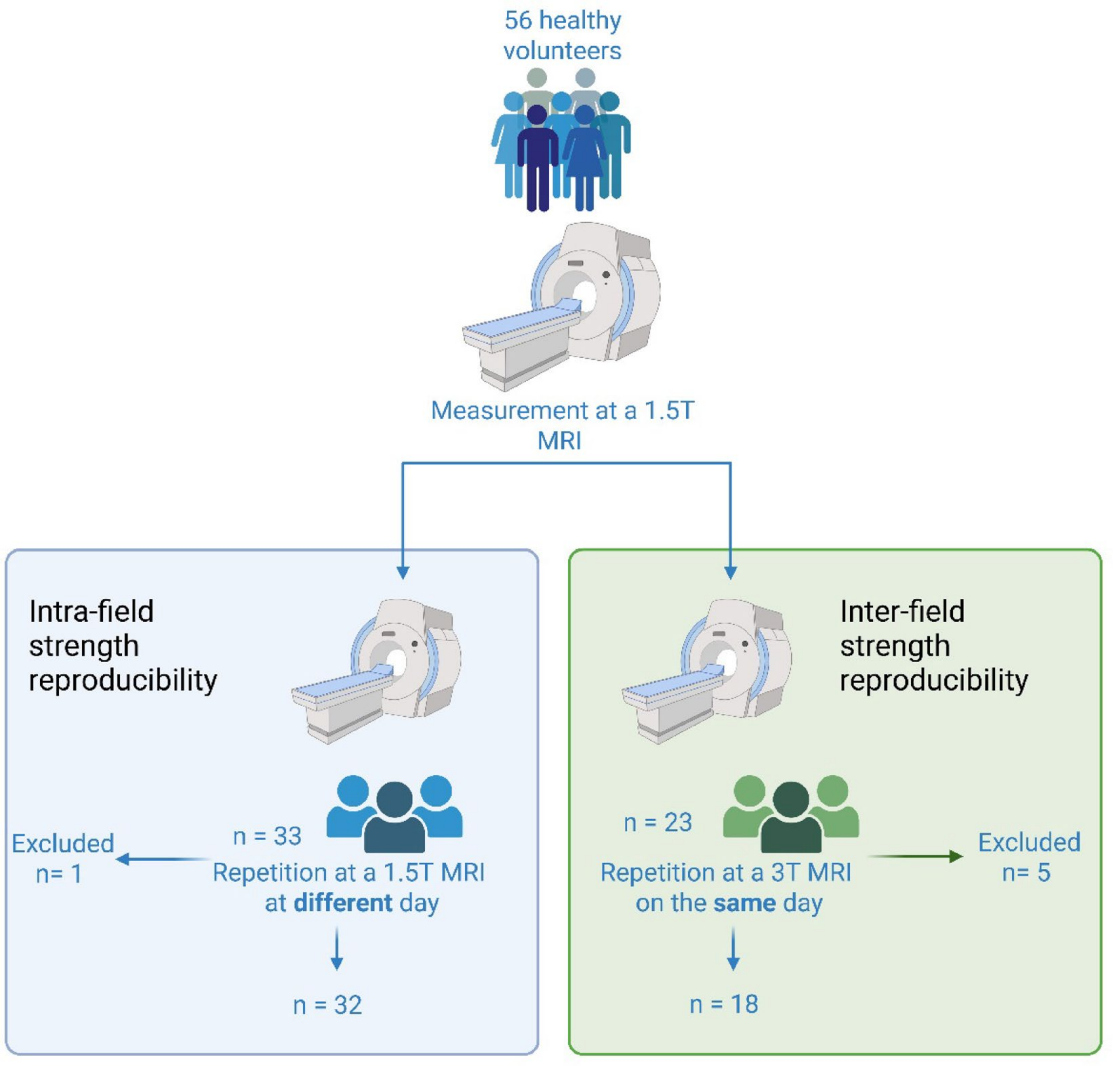
Study design.

For assessment of the inter-scanner reproducibility, the remaining 23 participants had a second scan on the same day, using a 3 T MRI system (Signa Premier, GE HealthCare, Solingen, Germany). The volunteers were randomly and depending on their availability selected to have their second scan at the 1.5 T or 3 T MRI scanner. The study design is displayed in Fig. 6.

The imaging protocol remained identical to the initial examination, including accelerated single-heartbeat (1RR) cine sequences (Sonic DL™, GE HealthCare) acquired both with breath-hold commands and in free breathing. The protocol covered a short-axis stack (SAX) as well as 2-, 3-, and 4-chamber views. A breath-hold cine sequence with three cardiac cycles per slice (3RR Sonic DL™) served as the reference for the volumetric measurements. This sequence has been previously validated, demonstrating no significant differences in volumetric results compared to conventional, non-DL-based cine sequences^11^. Detailed scan parameters for both sequences were as follows: Field of view = 34 × 34 cm^2^, slice thickness = 8 mm, frames/cardiac cycle = 30, TE = 1.2 ms. Differences in scan parameter could be found in the in-plane resolution with 1.7 × 1.5 mm^2^ vs. 1.9 × 1.9mm^2^, image pixel matrix with 200 × 224 vs. 180 × 160, TR with 3.3 ms vs. 3.2 ms and the acceleration factor with 6 vs. 12 for the 3RR Sonic DL and 1RR Sonic DL, respectively.

### Post-processing and volumetric analysis

Volumetric assessment was performed using commercial software (cvi42, version 5.16, Circle Cardiovascular Imaging Inc., Calgary, Canada). The software automatically delineated the endocardial and epicardial contours, with papillary muscles incorporated into the blood pool. For eliminating the potential biases and for ensuring consistency, only the fully automated segmentation without manual corrections was performed. Volumetric parameters for the left ventricle (LV), including end-diastolic volume (EDV), end-systolic volume (ESV), stroke volume (SV), ejection fraction (EF), and LV mass, were obtained.

### Subjective image quality assessment

Subjective image quality of the 18 volunteers measured at 1.5 T and 3 T was evaluated by two experienced radiologists, each with five years of expertise in CMR. An in-house developed software tool was employed to present the images in a randomized order, with blinding of the acquisition method (FB vs BH). Image quality was assessed using a five-point Likert scale (1 = lowest quality, non-diagnostic; 5 = excellent quality) for three specific criteria: absence of artefacts, image sharpness (i.e., absence of blurring) and image contrast. The evaluation results for each criterion were analysed separately, as well as combined as a mean value to provide an overall subjective quality score.

### Statistical analysis

Demographic characteristics and ventricular volumetric results of the study are expressed as medians with corresponding ranges (minimum to maximum), as the Shapiro–Wilk test for normality could not confirm that parameters follow a normal distribution.

The Wilcoxon matched-pairs signed-rank test was used to assess differences in volumetric results between the first and the second measurement of the reference, 1RR BH, and 1RR FB sequences as well as differences in quality between BH and FB sequences. Bland–Altman analysis was applied to show the absolute bias in volumetric results, along with standard deviation (SD) and the 95% limits of agreement (1.96*SD) of repeated examinations . The absolute difference of the volumetric parameter between both measurements is displayed as boxplots for all sequences (Reference, 1RR BH, 1RR FB). The Friedman test for multiple comparisons with Dunn’s post hoc test was used to identify significant differences between the groups.

In order to evaluate the consistency of all cine sequences between different methods, intra-class correlation coefficients (ICC) were calculated using two-way random-effects models based on volumetric data derived from the short-axis stack. Subjective image quality was expressed as the average per reader and as the overall mean score across both readers and all quality parameters. A *p*-value of < 0.05 was considered statistically significant. Statistical analyses were carried out using Stata 18.0 (Stata Corporation, College Station, TX, USA) and GraphPad Prism (Version 10.3.0; GraphPad Software, LLC, Boston, MA, USA).

## Data Availability

The datasets generated during and/or analysed during the current study are available from the corresponding author on reasonable request.

## Abbreviations

CMR: Cardiac magnetic resonance imaging
DL: Deep learning
FB: Free breathing
BH: Breath hold
EDV: End-diastolic volume
EF: Ejection fraction
ESV: End-systolic volume
LV: Left ventricle
MRI: Magnetic resonance imaging
RV: Right ventricle
SAX: Short-axis
SV: Stroke volume
2CH: 2-Chamber-view
3CH: 3-Chamber-view
4CH: 4-Chamber-view
ICCL: Intra-class-correlation coefficient
